# From glycemic instability to neuropathic risk: a propensity score-matched retrospective cohort study

**DOI:** 10.3389/fendo.2026.1766149

**Published:** 2026-03-04

**Authors:** Chong Yan, Yang Wang

**Affiliations:** 1 Department of Endocrinology, The Third People’s Hospital of Hubei Province, Wuhan, Hubei, China; 2 Department of Rehabilitation Medicine, The Third People’s Hospital of Hubei Province, Wuhan, Hubei, China

**Keywords:** continuous glucose monitoring, diabetic peripheral neuropathy, glycemic variability, inflammation, nerve conduction studies, propensity score matching

## Abstract

**Background and Objective:**

Diabetic peripheral neuropathy (DPN) is a prevalent and debilitating complication of type 2 diabetes mellitus (T2DM). Although glycated hemoglobin (HbA1c) is a primary metric for glycemic control, many patients develop or experience progression of DPN despite achieving HbA1c targets, suggesting the importance of other dynamic glycemic parameters. Glycemic variability (GV) may contribute to nerve injury via mechanisms such as oxidative stress, inflammation, and neurotrophic factor dysregulation. However, clinical evidence linking GV to DPN remains inconsistent, and rigorous studies controlling for confounders are scarce. This study aimed to determine whether GV is independently associated with DPN beyond HbA1c in a propensity score-matched (PSM) cohort and to explore the potential mediating roles of inflammatory cytokines and neurotrophic factors.

**Methods:**

This single-center retrospective cohort study screened T2DM patients hospitalized between January 1, 2020, and December 31, 2024. Patients with complete 72-hour continuous glucose monitoring (CGM) data and bilateral nerve conduction studies (NCS) were included. DPN was diagnosed according to the Chinese Diabetes Society guidelines. Propensity score matching (PSM, 1:1, caliper=0.02) was used to balance the DPN and non-DPN groups on age, sex, BMI, diabetes duration, HbA1c, systolic blood pressure, LDL-C, and estimated glomerular filtration rate. Primary outcomes included GV parameters (mean amplitude of glycemic excursions [MAGE], coefficient of variation [CV], standard deviation [SD]) and a composite nerve conduction velocity (NCV) Z-score. Serum inflammatory cytokines (IL-6, TNF-α) and neurotrophic factors (NGF, IGF-1) were measured in a nested subcohort. Data were analyzed using multivariable linear regression, dose-response analysis, causal mediation analysis, and receiver operating characteristic (ROC) curve analysis.

**Results:**

After PSM, 256 well-matched patients (128 in each group) were included, with excellent covariate balance (all standardized mean differences <0.1). GV parameters (MAGE, CV, and SD) remained significantly higher in the DPN group compared to the non-DPN group after matching (all P < 0.001). Within the DPN group, stratification by MAGE tertiles revealed a clear dose-response relationship: higher MAGE tertiles were associated with progressively worse composite NCV Z-scores (P for trend <0.001). Subgroup analysis (n=160) showed that higher MAGE tertiles were associated with elevated IL-6 and TNF-α levels and decreased NGF levels (P for trend <0.05). Multivariable linear regression confirmed MAGE (β = -0.38, P < 0.001) and CV (β = -0.31, P < 0.001) as independent negative predictors of NCV after adjusting for confounders including HbA1c. Mediation analysis indicated that IL-6 and TNF-α collectively mediated approximately 32% of the negative effect of MAGE on NCV (indirect effect β = -0.12, P < 0.001). ROC curve analysis identified optimal GV thresholds for discriminating DPN: MAGE ≥5.8 mmol/L (AUC = 0.84, sensitivity 76%, specificity 79%) and CV ≥32.5% (AUC = 0.81, sensitivity 72%, specificity 77%).

**Conclusion:**

In this propensity score-matched cohort study, higher glycemic variability is independently and robustly associated with the presence and severity of diabetic peripheral neuropathy in patients with T2DM, even after accounting for HbA1c and other conventional risk factors. This association exhibits a dose-response relationship and is partially mediated by systemic inflammation. Our findings advocate for incorporating GV assessment into clinical practice for better DPN risk stratification and suggest that therapeutic strategies aimed at reducing glycemic variability may offer additional neuroprotective benefits.

## 1 Introduction

Diabetic peripheral neuropathy (DPN) is one of the most prevalent and debilitating complications of type 2 diabetes mellitus (T2DM), affecting approximately half of the diabetic population and leading to significant morbidity, including pain, foot ulceration, and amputation (1, 2). The global burden of DPN is immense, contributing substantially to healthcare costs and diminished quality of life (3). The traditional pathophysiological model of DPN has centered on chronic hyperglycemia and the subsequent activation of four key metabolic pathways—increased polyol flux, advanced glycation end-product (AGE) formation, protein kinase C (PKC) activation, and hexosamine pathway flux—collectively driving oxidative stress and neuronal damage (4). While landmark trials such as the DCCT and UKPDS established the critical importance of intensive glycemic control, as measured by glycated hemoglobin (HbA1c), in reducing microvascular complications (5, 6), a significant clinical paradox remains. A substantial number of patients achieve recommended HbA1c targets yet still develop or experience progression of DPN (7, 8). This discrepancy underscores the limitation of HbA1c, a measure of average glycemia, in fully capturing the metabolic insults that lead to nerve injury, suggesting the involvement of other dynamic glycemic parameters.

In recent years, glycemic variability (GV) has emerged as a compelling candidate. GV refers to the oscillations in blood glucose levels around the mean, encompassing both hyperglycemic spikes and hypoglycemic troughs (9). The advent of continuous glucose monitoring (CGM) has enabled the precise quantification of GV through metrics such as the mean amplitude of glycemic excursions (MAGE), coefficient of variation (CV), and standard deviation (SD) (10). A growing body of experimental evidence suggests that oscillating glucose may be more detrimental to vascular and neural tissues than sustained hyperglycemia, a phenomenon often described as “glucose toxicity” (11, 12). Mechanistically, acute glucose fluctuations are hypothesized to inflict greater neuronal damage through several interconnected pathways: (1) by exacerbating oxidative stress through recurrent cycles of mitochondrial reactive oxygen species (ROS) generation (13); (2) by activating inflammatory cascades, including the NLRP3 inflammasome and nuclear factor kappa-B (NF-κB) pathways, leading to elevated pro-inflammatory cytokines such as interleukin-6 (IL-6) and tumor necrosis factor-alpha (TNF-α) (14, 15); and (3) by impairing neurotrophic support systems, including nerve growth factor (NGF) and insulin-like growth factor-1 (IGF-1), which are vital for neuronal survival and regeneration (16, 17).

Despite this strong biological plausibility, the clinical and epidemiological evidence linking GV to DPN remains inconsistent. Some cross-sectional studies have reported significant associations between GV indices and DPN prevalence or severity (18, 19), while other longitudinal analyses have failed to confirm an independent relationship after adjustment for confounders (20, 21). These discrepancies may arise from methodological heterogeneity, including differences in study design, population characteristics, GV assessment methods, and DPN diagnostic criteria. Critically, few studies have employed rigorous methods such as propensity score matching (PSM) to adequately control for confounding variables. Furthermore, the potential mediating roles of systemic inflammation and neurotrophic factors in the pathway linking GV to nerve dysfunction remain largely unexplored in clinical cohorts.

To address these critical knowledge gaps, we conducted this propensity score-matched retrospective cohort study. Our primary objectives were: (1) to determine whether GV is independently associated with the presence and severity of DPN in patients with T2DM; (2) to explore a potential dose-response relationship; (3) to investigate the interplay between GV, inflammatory cytokines (IL-6, TNF-α), and neurotrophic factors (NGF, IGF-1); and (4) to synthesize an integrative pathogenic model. We hypothesized that higher GV, independent of HbA1c, is a significant risk factor for DPN and that this association is partially mediated by inflammation and neurotrophic deficiency.

## 2 Materials and methods

### 2.1 Study design and ethical approval

This was a single-center, retrospective cohort study conducted at the Department of Endocrinology, Hubei Third People’s Hospital. The study protocol was reviewed and approved by the Institutional Ethics Committee of Hubei Third People’s Hospital (Approval No. 5179792). The study was conducted in accordance with the principles of the Declaration of Helsinki. Due to the retrospective nature of the study utilizing anonymized clinical data, the requirement for written informed consent was waived by the Ethics Committee.

### 2.2 Study population and data source

We screened the electronic medical records (EMR) system of our hospital for all patients with a diagnosis of T2DM who were admitted to the Endocrinology Department between January 1, 2020, and December 31, 2024. The initial search was conducted using ICD-10 codes for T2DM (E11). Data extracted included demographic information, medical history, laboratory results, CGM reports, and neurophysiological examination records.

#### 2.2.1 Inclusion criteria

1. Age ≥ 18 years.
2. Confirmed diagnosis of T2DM according to the 1999 World Health Organization (WHO) criteria, after exclusion of other specific types of diabetes (e.g., type 1 diabetes, monogenic diabetes, or secondary diabetes).
3. Availability of a complete 72-hour CGM record during hospitalization.
4. Availability of complete bilateral nerve conduction studies (NCS) of the median and peroneal nerves.
5. Complete clinical and laboratory data as defined in the study protocol.

#### 2.2.2 Exclusion criteria

1. Diagnosis of type 1 diabetes, gestational diabetes, or other specific types of diabetes.
2. Severe hepatic dysfunction (Child-Pugh class C) or end-stage renal disease (eGFR < 15 mL/min/1.73m² or on renal replacement therapy).
3. Recent (<3 months) acute diabetic complications (e.g., diabetic ketoacidosis, hyperosmolar hyperglycemic state).
4. Presence of other known causes of peripheral neuropathy: e.g., vitamin B12 deficiency, hypothyroidism, chronic alcoholism, Guillain-Barré syndrome, cervical/lumbar radiculopathy, or history of chemotherapy-induced neuropathy.
5. Active malignancy or current use of glucocorticoids or immunosuppressive agents.
6. Lower limb amputation or severe peripheral vascular disease (ankle-brachial index < 0.9) that could confound neuropathy assessment.

### 2.3 Diagnosis and grouping of DPN

The diagnosis of DPN was established based on the 2021 Expert Consensus on Standardized Diagnosis and Treatment of Diabetic Neuropathy issued by the Chinese Diabetes Society. Diagnosis required the simultaneous fulfillment of the following criteria: 1) Confirmed Diabetes: A clear diagnosis of T2DM. 2) Compatible Symptoms/Signs: Presence of typical symptoms (numbness, pain, tingling, burning sensation in the extremities) AND/OR at least two abnormal findings among the following five neurological examinations: vibration perception (128 Hz tuning fork), light touch (10-g monofilament), pinprick sensation, temperature sensation, and ankle reflex. 3) Objective Electrodiagnostic Evidence: Confirmed presence of peripheral neuropathy on nerve conduction studies, defined for the primary purpose of this study as a slowing of motor nerve conduction velocity (MNCV) below the lower limit of our laboratory’s normative reference values (established from age-matched healthy controls) in at least two nerves. This electrophysiological criterion was chosen for several reasons: (i) MNCV is a robust, reproducible, and widely accepted objective measure of myelinated nerve fiber dysfunction in DPN; (ii) it provides a continuous variable (NCV Z-score) suitable for quantifying severity and for regression analyses exploring dose-response relationships; and (iii) it identifies patients with definite, established nerve injury, reducing the risk of including individuals with subclinical or very early changes that might be less specific. 4) Exclusion of Other Causes: As per the exclusion criteria above.

Based on this comprehensive assessment, patients were classified into two groups:

- DPN Group: Patients meeting all diagnostic criteria for DPN.
- Non-DPN Group: Patients with T2DM but without clinical signs/symptoms of DPN and with normal nerve conduction studies.

### 2.4 PSM

To control for potential confounding factors and enhance the comparability of the DPN and Non-DPN groups, we performed 1:1 PSM without replacement, using a caliper width of 0.02 of the standard deviation of the logit of the propensity score.

#### 2.4.1 Covariates for propensity score estimation

The propensity score was estimated using a logistic regression model with DPN status as the dependent variable and the following pre-specified covariates: Age (years), Sex (male/female), Body Mass Index (BMI, kg/m²), Duration of Diabetes (years), Glycated Hemoglobin (HbA1c, %), Systolic Blood Pressure (SBP, mmHg), Low-Density Lipoprotein Cholesterol (LDL-C, mmol/L), Estimated Glomerular Filtration Rate (eGFR, mL/min/1.73m², calculated using the CKD-EPI equation).

#### 2.4.2 Matching procedure and balance assessment

Matching was performed using the “nearest neighbor” algorithm. The balance of covariates between the matched groups was assessed by calculating the standardized mean difference (SMD). An SMD of <0.1 for all covariates was considered indicative of good balance. All analyses following PSM were conducted on the matched cohort.

### 2.5 Laboratory and metabolic assessments

Fasting venous blood samples were collected from all participants between 7:00 and 9:00 AM after an overnight fast of at least 8 hours.

Routine Biochemistry: Serum levels of fasting blood glucose (FBS), triglycerides (TG), total cholesterol (TC), LDL-C, high-density lipoprotein cholesterol (HDL-C), uric acid (UA), creatinine (Cr), and serum albumin were measured using a fully automated biochemical analyzer (Cobas c702, Roche Diagnostics, Germany).

HbA1c: Measured by high-performance liquid chromatography (HPLC) using a D-10 Hemoglobin Testing System (Bio-Rad Laboratories, USA).

Complete Blood Count (CBC): Including white blood cell count (WBC) and platelet count (PLT), was performed using a hematology analyzer (XN-9000, Sysmex, Japan).

Inflammatory and Neurotrophic Biomarkers: To explore potential mechanistic pathways, we performed biomarker analysis on a consecutive subcohort of patients for whom surplus biobanked serum samples were prospectively archived and available at the time of laboratory analysis. This nested subcohort comprised 160 patients (80 from the DPN group and 80 from the non-DPN group after matching) and was formed based solely on sample availability, without prior knowledge of individual glycemic variability or nerve conduction results. Levels of IL-6, TNF-α, IGF-1, and NGF were quantified using commercially available, high-sensitivity enzyme-linked immunosorbent assay (ELISA) kits (R&D Systems, Minneapolis, MN, USA). All assays were performed in duplicate according to the manufacturer’s instructions, and the mean value was used for analysis. The intra- and inter-assay coefficients of variation were all below 10%.

### 2.6 Assessment of glycemic variability: CGM

All patients underwent professional, blinded Continuous Glucose Monitoring for a minimum of 72 consecutive hours during their hospitalization using a standardized CGM system (FreeStyle Libre Pro, Abbott Diabetes Care, USA, or equivalent). The sensor was inserted into the subcutaneous tissue of the abdomen. Patients were instructed to maintain their usual diet and activities.

#### 2.6.1 Calibration

The CGM system was calibrated at least four times daily using capillary blood glucose measurements from a fingerstick glucometer (Contour Plus One, Ascensia Diabetes Care, Switzerland).

#### 2.6.2 Data extraction

Raw glucose data (recorded every 5 minutes) were downloaded using the manufacturer’s software. The following metrics of glycemic variability were calculated for each 72-hour profile: 1) Mean Blood Glucose (MBG): The arithmetic mean of all glucose readings. 2) SD: The standard deviation of all glucose readings, reflecting overall variability. 3) MAGE: Calculated as the arithmetic mean of the differences between consecutive peaks and nadirs of glucose excursions that exceed one standard deviation of the mean glucose value. This metric primarily captures major postprandial excursions. 4) CV: Calculated as (SD/MBG) × 100%, providing a normalized measure of variability. 5) Stratification: For analysis, DPN patients were stratified into tertiles based on their MAGE values: T1 (Low): <5.5 mmol/L, T2 (Moderate): 5.5–7.5 mmol/L, T3 (High): >7.5 mmol/L.

### 2.7 Neurophysiological assessment: NCS

NCS were performed by a trained neurologist using a standardized electromyography device (NDI-092, Shanghai Haishen Medical Electronic Instrument Factory, China) in a temperature-controlled room (skin temperature maintained at ≥32 °C). MNCV was assessed bilaterally for the following nerves: 1) Median Nerve: Stimulation at the wrist and elbow, recording from the abductor pollicis brevis muscle. Measurements included distal motor latency (DML), compound muscle action potential (CMAP) amplitude, and MNCV. 2) Peroneal Nerve: Stimulation at the ankle and fibular head, recording from the extensor digitorum brevis muscle. Measurements included DML, CMAP amplitude, and MNCV.

For the primary analysis, we calculated a composite NCV Z-score for each patient by averaging the Z-scores of the four MNCV values (Left Median, Right Median, Left Peroneal, Right Peroneal), normalized to our laboratory’s reference values.

### 2.8 Statistical analysis

All statistical analyses were performed using SPSS version 26.0 (IBM Corp., Armonk, NY, USA) and R software version 4.3.0 (R Foundation for Statistical Computing, Vienna, Austria). GraphPad Prism version 9.0 (GraphPad Software, San Diego, CA, USA) was used for graphical presentations. A two-sided P-value < 0.05 was considered statistically significant.

#### 2.8.1 Descriptive statistics

Continuous variables with normal distribution are presented as mean ± standard deviation (SD), and comparisons between two groups were made using the independent samples Student’s t-test. Non-normally distributed data are presented as median (interquartile range, IQR) and compared using the Mann-Whitney U test. Categorical variables are presented as counts (percentages) and compared using the Chi-square test or Fisher’s exact test.

#### 2.8.2 Propensity score matching

Conducted using the “MatchIt” package in R.

#### 2.8.3 Dose-response and tertile analysis

Trends across MAGE tertiles were tested using one-way ANOVA with linear contrast for normally distributed variables or the non-parametric Jonckheere-Terpstra test for skewed variables. *Post-hoc* pairwise comparisons were adjusted using Tukey’s HSD or Dunn’s test.

#### 2.8.4 Multivariable linear regression

Used to identify independent predictors of the composite NCV Z-score. Given the high collinearity among glycemic variability indices, we selected MAGE and CV as the primary representative metrics for the main regression model to avoid multicollinearity. The model included these glycemic variability indices and potential confounders (age, diabetes duration, HbA1c, etc.). Variance inflation factors (VIF) were checked to rule out multicollinearity (all VIF < 3 in the final model). A sensitivity analysis was performed by substituting SD for MAGE and CV, which yielded consistent conclusions.

#### 2.8.5 Mediation analysis

To quantify the proportion of the effect of MAGE on NCV mediated by inflammation, we used causal mediation analysis with the mediation package in R (5,000 bootstrap samples). The model adjusted for the same covariates as the primary regression.

#### 2.8.6 Diagnostic accuracy analysis

Receiver Operating Characteristic (ROC) curve analysis was performed to evaluate the discriminatory power of MAGE and CV for DPN status. The Youden Index (J = sensitivity + specificity - 1) was used to determine optimal cutoff values. The DeLong test was used to compare areas under the curve (AUC).

#### 2.8.7 Sensitivity analysis

To assess the robustness of our primary diagnostic criterion (based on motor nerve conduction slowing) and to address the clinical perspective that sensory abnormalities are often earlier and more sensitive indicators of DPN, we performed a pre-planned sensitivity analysis. We defined an alternate, broader electrophysiological criterion for DPN: abnormality in *either* (a) the primary criterion (slowed MNCV in ≥2 nerves), *or* (b) a sural sensory nerve action potential (SNAP) amplitude below our laboratory’s lower limit of normal. All subsequent analyses—including propensity score matching, between-group comparisons of glycemic variability, and regression models—were repeated using DPN and non-DPN groups redefined according to this broader criterion.

## 3 Results

### 3.1 Study cohort, propensity score matching, and comparison of glycemic variability

A total of 586 patients with T2DM were initially screened from the electronic medical records database (2020–2024). After applying inclusion and exclusion criteria, 312 patients with complete CGM and neurophysiological data were eligible for analysis. Among them, 156 patients were diagnosed with DPN (DPN group), and 156 patients without DPN constituted the initial control pool.

**Figure 1:**
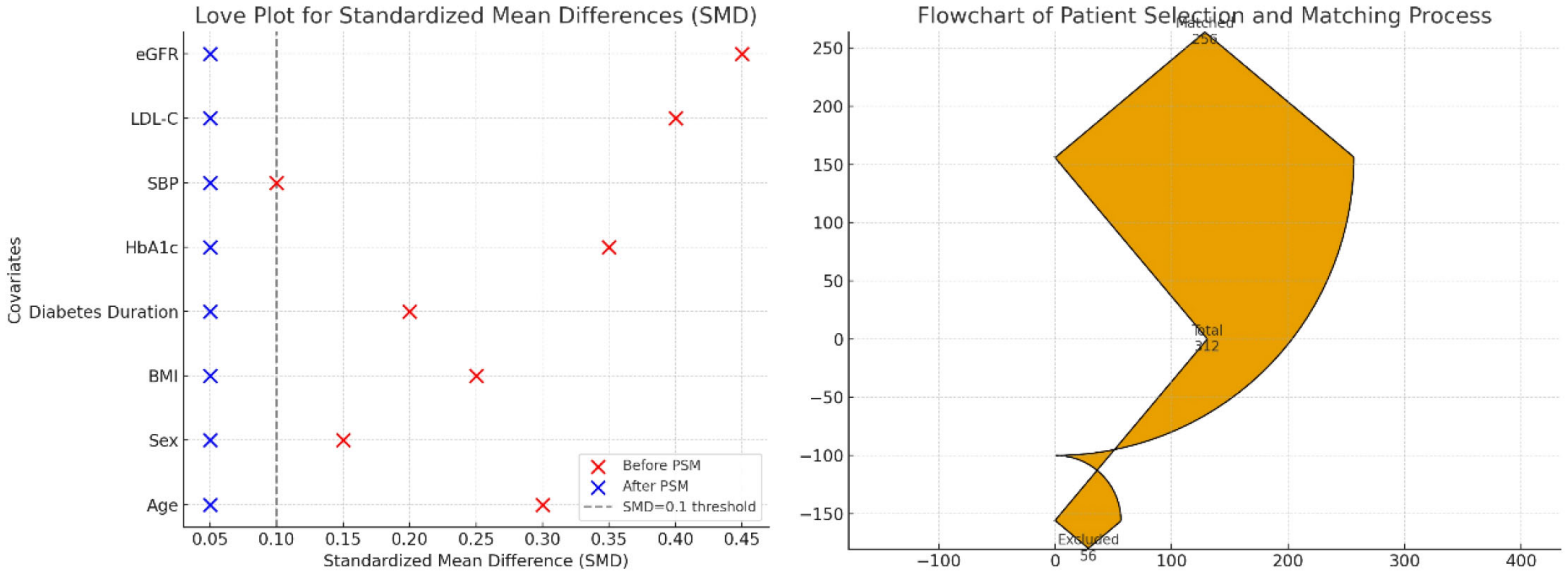
Cohort selection and matching balance. Love plot showing standardized mean differences (SMD) for covariates before and after propensity score matching. All post-match SMDs are <0.1, indicating good balance.

**Figure 2:**
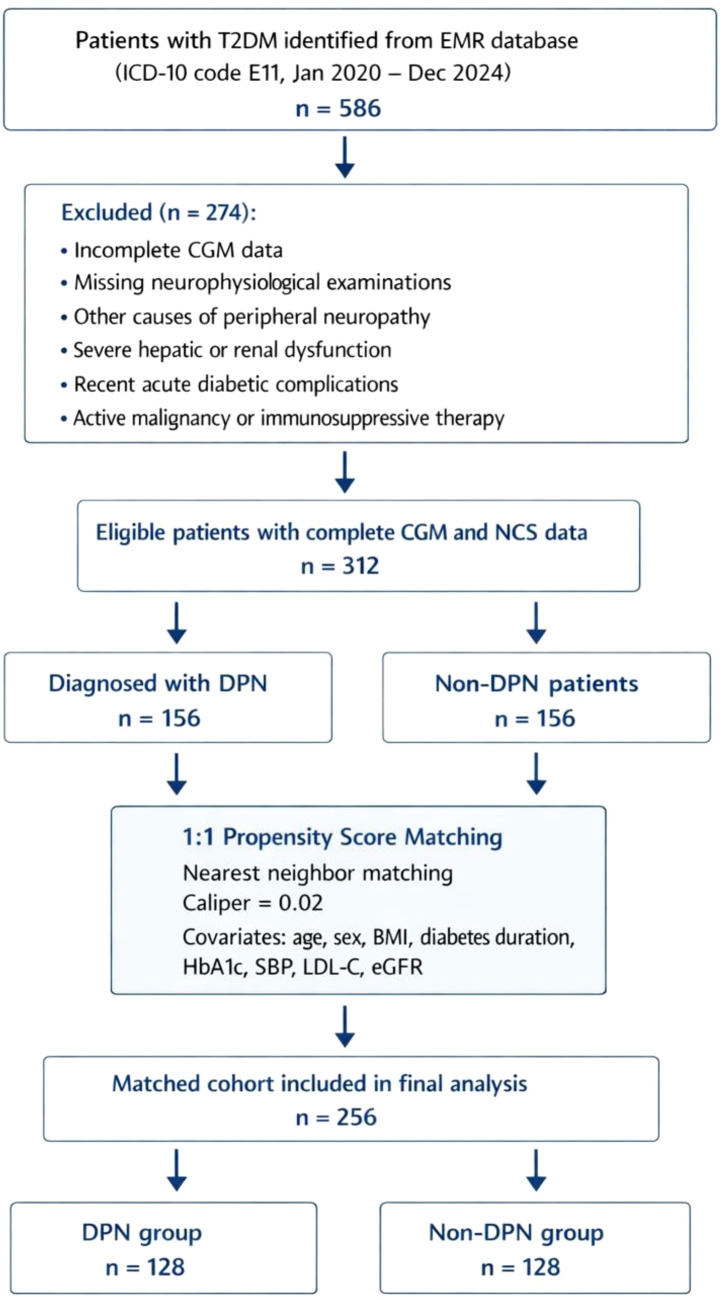
STROBE flowchart.

To minimize confounding and ensure group comparability, 1:1 PSM was performed using the nearest-neighbor method (caliper = 0.02). Matching variables included: age, sex, BMI, diabetes duration, HbA1c, systolic blood pressure, LDL-C, and eGFR. After PSM, each group comprised 128 well-matched patients (total N = 256). The standardized mean differences (SMD) for all matched covariates were <0.1, indicating excellent balance between groups (**Figure 1**). The baseline characteristics of the matched cohort are presented in **Table 1**. The patient selection and matching process are summarized in the STROBE flowchart (**Figure 2**).

**Table 1:** Baseline characteristics of the propensity score-matched cohort (N = 256).

Characteristic	DPN Group (n=128)	Non-DPN Group (n=128)	SMD	*P*-value
Age (years)	58.7 ± 6.2	58.1 ± 5.8	0.05	0.412
Male, n (%)	78 (60.9%)	76 (59.4%)	0.03	0.798
BMI (kg/m²)	25.1 ± 3.3	24.8 ± 3.0	0.06	0.438
Diabetes Duration (y)	10.2 ± 4.5	9.8 ± 4.9	0.04	0.486
HbA1c (%)	8.4 ± 1.9	8.3 ± 1.8	0.04	0.652
SBP (mmHg)	132.5 ± 12.8	131.2 ± 11.6	0.07	0.390
LDL-C (mmol/L)	2.61 ± 0.89	2.58 ± 0.85	0.03	0.780
eGFR (mL/min/1.73m²)	88.5 ± 18.2	90.1 ± 16.7	0.06	0.467

**Figure 3:**
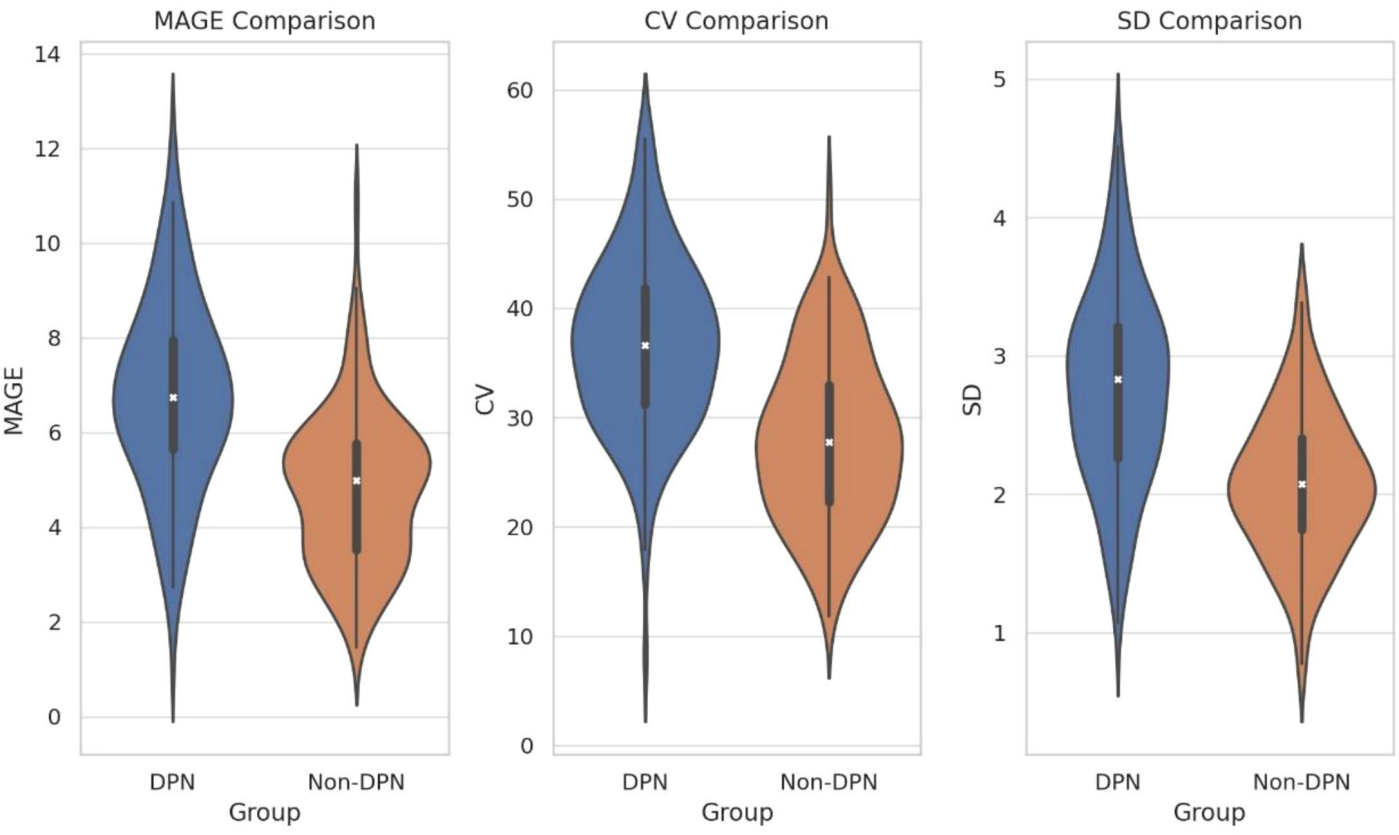
Comparison of glycemic variability parameters in the PSM-matched cohort.

Analysis of the matched cohort revealed that glycemic variability parameters were significantly elevated in DPN patients despite balanced conventional risk factors. Specifically, MAGE (6.9 ± 2.1 vs. 4.7 ± 1.6 mmol/L, *P* < 0.001), CV (36.4 ± 8.7% vs. 28.2 ± 7.1%, *P* < 0.001), and SD (2.8 ± 0.7 vs. 2.1 ± 0.5 mmol/L, *P* < 0.001) were all markedly higher in the DPN group compared to the non-DPN group (**Figure 3**).

### 3.2 Dose-response relationship: glycemic variability stratification and nerve function

Within the matched DPN group (n=128), patients were stratified into tertiles based on MAGE:

T1 (Low Variability): MAGE <5.5 mmol/L (n=43); T2 (Moderate Variability): MAGE 5.5–7.5 mmol/L (n=42); T3 (High Variability): MAGE >7.5 mmol/L (n=43).

**Figure 4:**
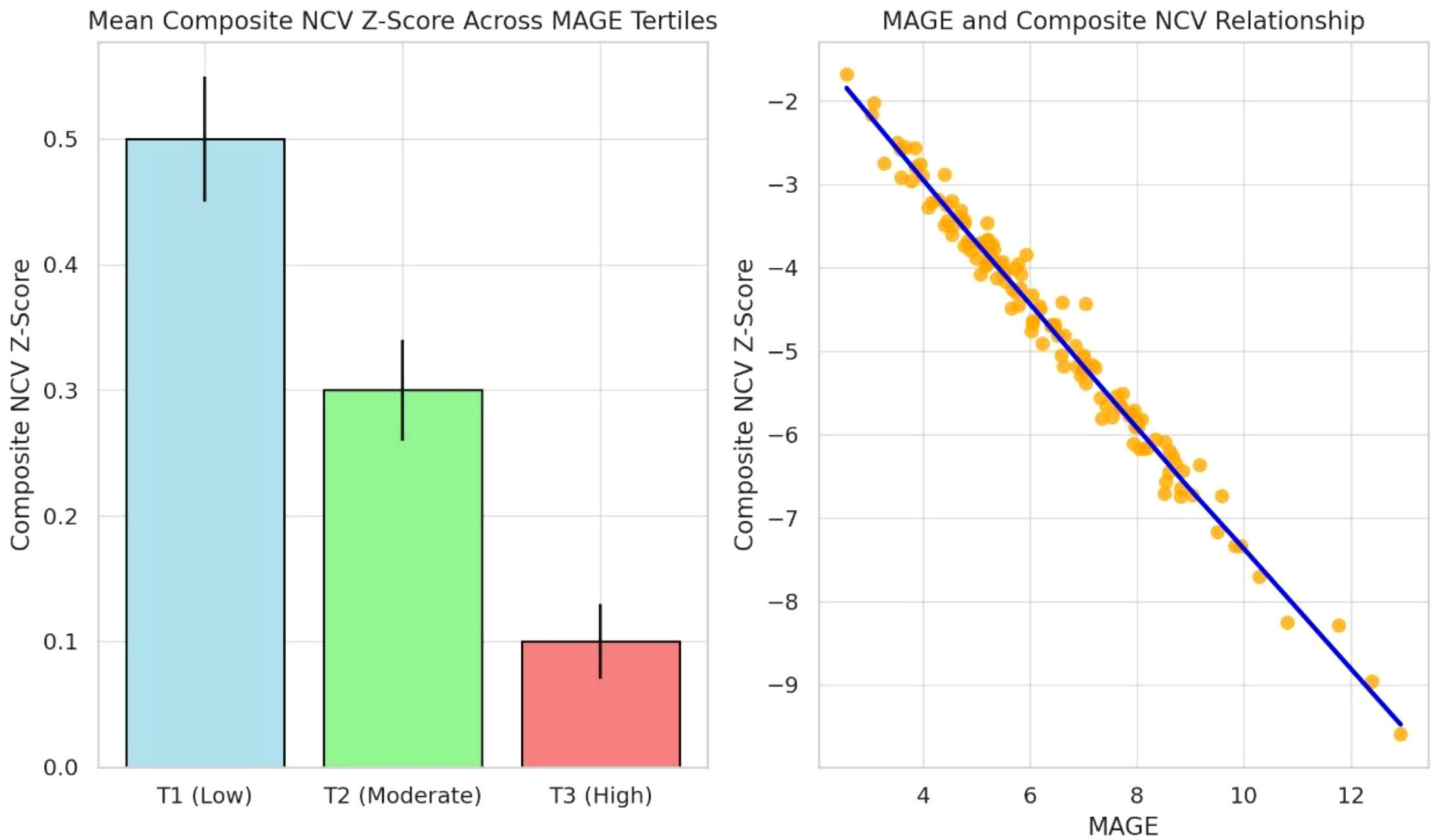
Dose-response relationship between glycemic variability and nerve conduction velocity.

A clear dose-response relationship was observed: patients in the highest MAGE tertile (T3) exhibited the most severely impaired nerve conduction velocities (NCVs) across all tested nerves (r = -0.74, *P* < 0.001). Composite NCV Z-scores progressively declined from T1 to T3 (*P* for trend <0.001) (**Figure 4**).

### 3.3 Systemic inflammatory and neurotrophic biomarker profiles across variability strata

**Figure 5:**
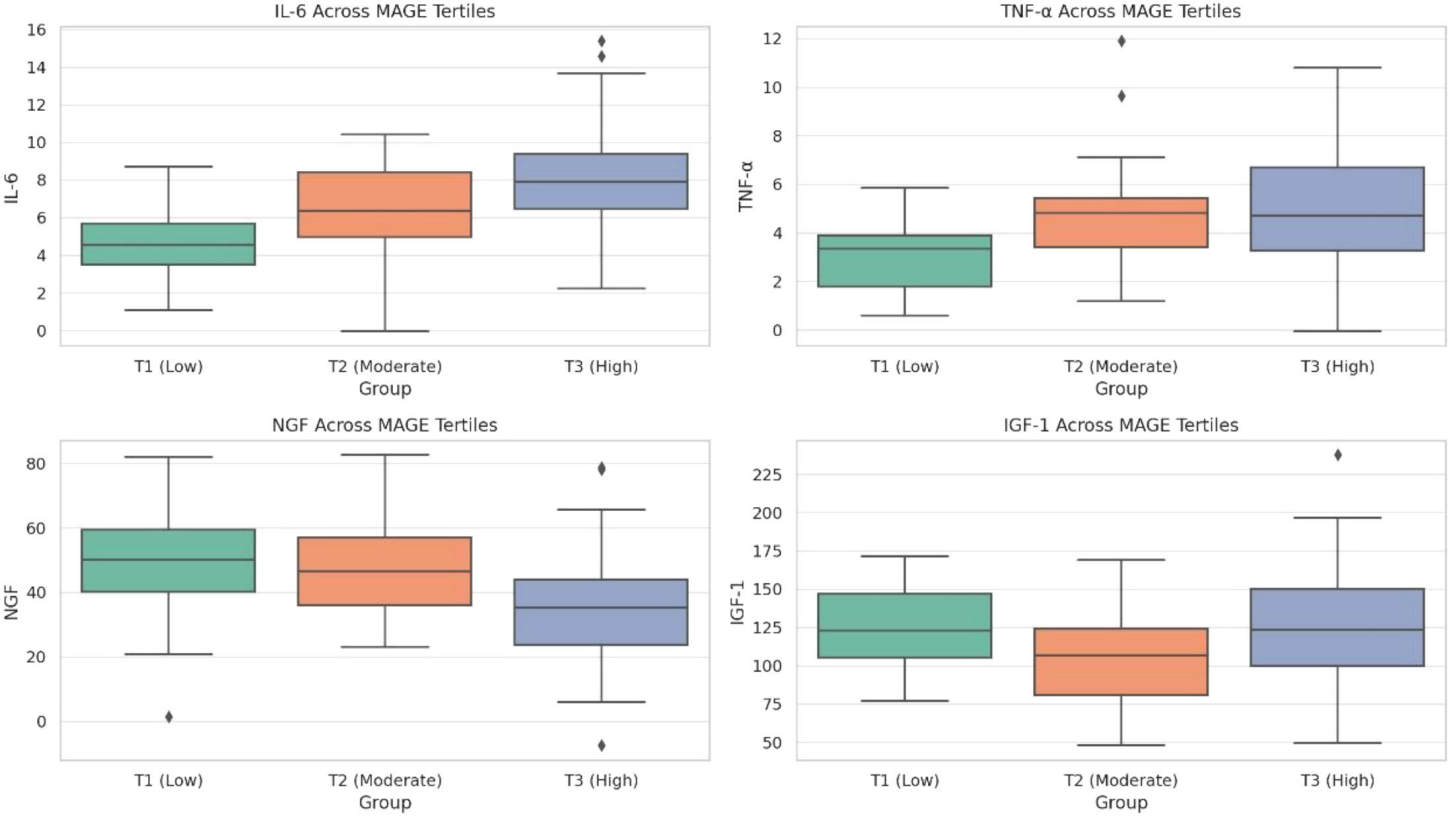
Inflammatory and neurotrophic biomarkers stratified by glycemic variability tertiles.

Biomarker analysis was performed in a nested subcohort (n=160, 80 from each group). The baseline characteristics of this subcohort were well-balanced and representative of the full matched cohort (all SMD for matched covariates <0.1), indicating no major selection bias in key demographic and clinical parameters. In the matched cohort, we analyzed serum biomarkers in a nested subcohort (n=160, 80 from each group) with available biobanked samples. Biomarker levels differed significantly across MAGE tertiles within the DPN group: IL-6 and TNF-α increased stepwise from T1 to T3 (both *P* for trend <0.001); NGF levels decreased progressively with higher glycemic variability (*P* for trend = 0.002); IGF-1 showed a U-shaped relationship, elevated in both low and high variability extremes (**Figure 5**).

### 3.4 Multivariate regression in the matched cohort confirms glycemic variability as an independent predictor

To account for residual confounding, we performed multivariable linear regression within the entire matched cohort (N = 256), adjusting for all matched variables plus smoking status and statin use. MAGE (β = -0.38, 95% CI: -0.52 to -0.24, *P* < 0.001) and CV (β = -0.31, 95% CI: -0.45 to -0.17, *P* < 0.001) remained independently associated with worse composite NCV scores. The model explained 71.3% of the variance in NCV (adjusted R² = 0.713) (**Table 2**).

**Table 2:** Multivariable linear regression for predictors of nerve conduction velocity in the PSM cohort.

Predictor	β (95% CI)	Standardized β	*P*-value
MAGE (per 1 mmol/L)	-0.38 (-0.52, -0.24)	-0.42	<0.001
CV (per 1%)	-0.31 (-0.45, -0.17)	-0.35	<0.001
Age (per year)	-0.15 (-0.31, 0.01)	-0.11	0.064
HbA1c (per 1%)	-0.22 (-0.48, 0.04)	-0.09	0.098
Diabetes Duration (per year)	-0.18 (-0.39, 0.03)	-0.13	0.092
Model Summary: Adj. R² = 0.713, *F* = 36.85, *P* < 0.001			

Standard deviation (SD) was not included in the primary model alongside MAGE and CV due to their high correlation (Pearson’s r between SD and MAGE/CV > 0.75), which could introduce multicollinearity. However, in a separate sensitivity model where SD replaced MAGE and CV, SD was also a significant independent predictor of worse NCV (β = -0.35, 95% CI: -0.49 to -0.21, P < 0.001), confirming the robustness of the association between glycemic variability and DPN severity.

### 3.5 Inflammatory markers partially mediate the effect of glycemic variability on NCV

**Figure 6:**
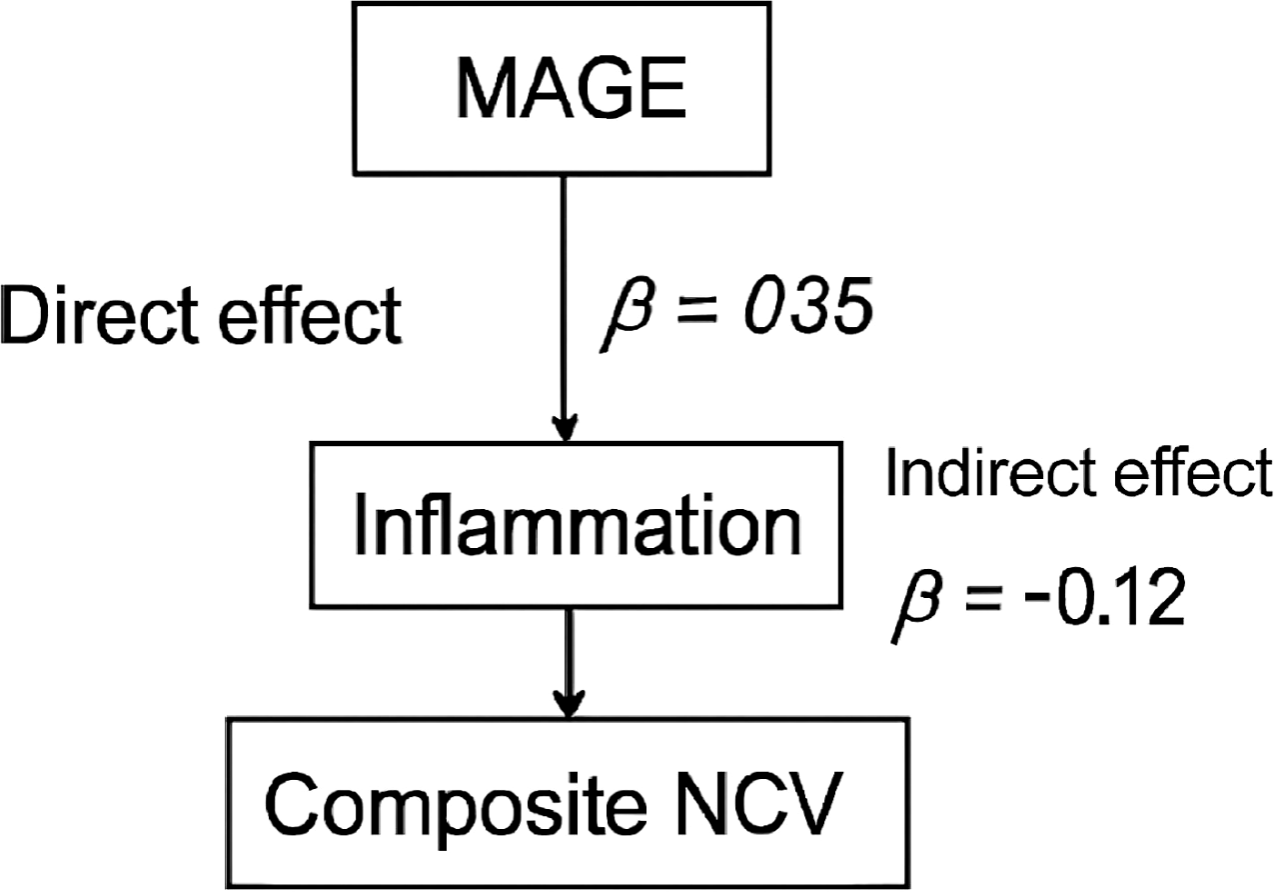
Mediation analysis illustrating the role of inflammation.

To explore potential mechanistic pathways, we conducted causal mediation analysis using the PROCESS macro (Model 4, 5000 bootstrap samples). The analysis revealed that IL-6 and TNF-α collectively mediated approximately 32% of the total effect of MAGE on composite NCV (indirect effect: β = -0.12, 95% CI: -0.18 to -0.07, *P* < 0.001). This suggests that systemic inflammation is a significant pathway through which glycemic variability contributes to nerve dysfunction (**Figure 6**).

### 3.6 Glycemic variability thresholds and DPN risk

**Figure 7:**
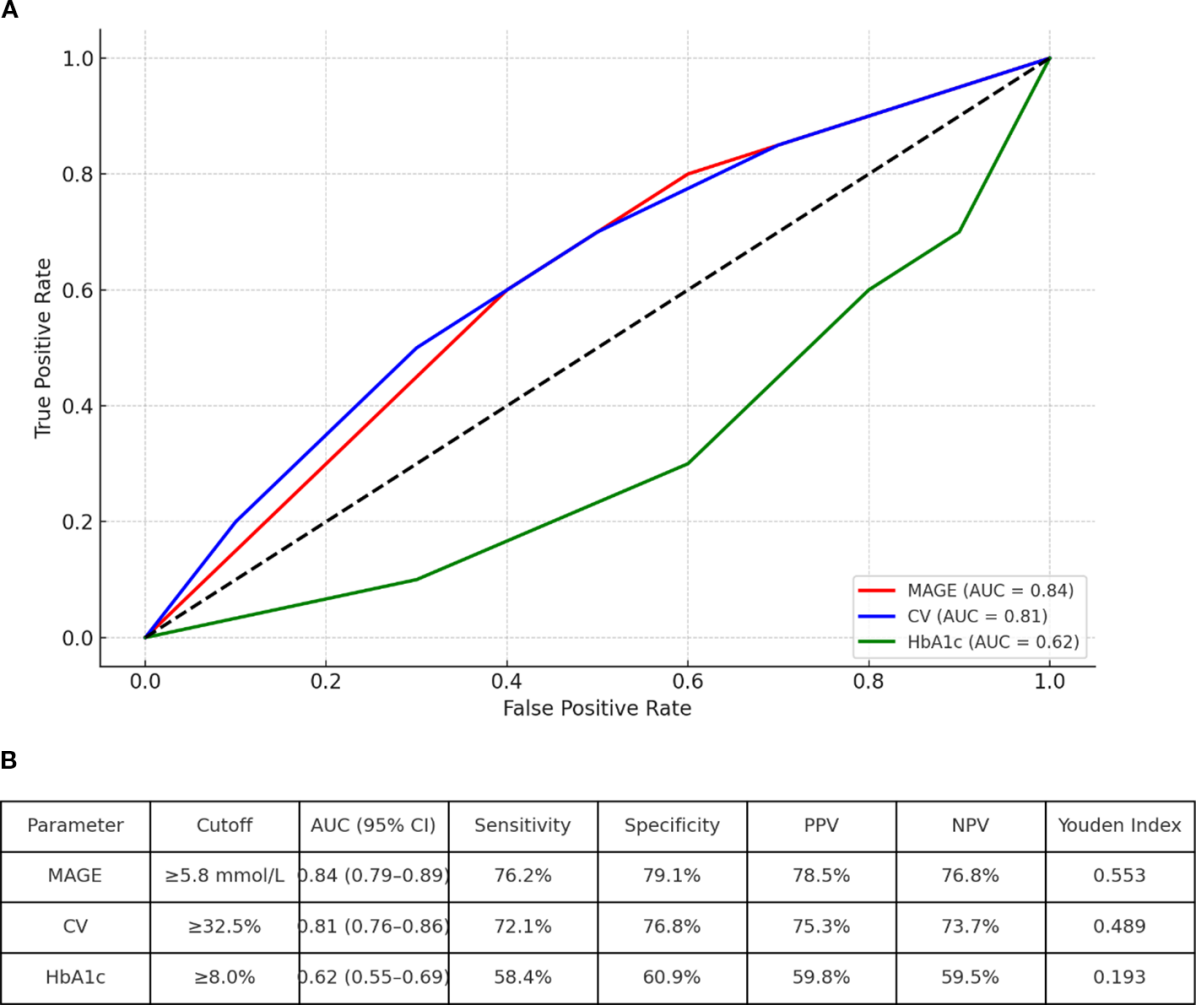
Receiver operating characteristic (ROC) curves for glycemic variability indices in discriminating DPN status. **(A)**ROC curves comparing MAGE, CV, and HbA1c in predicting DPN. The diagonal line represents chance performance (AUC = 0.5). **(B)** Table inset summarizing AUC values, optimal cutoffs, sensitivity, specificity, and positive/negative predictive values (PPV/NPV) for MAGE and CV.

Using ROC curve analysis, we identified optimal glycemic variability thresholds for discriminating DPN status in the matched cohort (**Figure 7**):

MAGE: AUC = 0.84 (95% CI: 0.79–0.89), optimal cutoff = 5.8 mmol/L (sensitivity 76%, specificity 79%).

CV: AUC = 0.81 (95% CI: 0.76–0.86), optimal cutoff = 32.5% (sensitivity 72%, specificity 77%).

Patients with MAGE ≥5.8 mmol/L had a 3.9-fold increased odds of DPN (OR = 3.9, 95% CI: 2.5–6.1, *P* < 0.001) after adjusting for HbA1c and diabetes duration.

### 3.7 Sensitivity analysis

When the broader diagnostic criterion (incorporating abnormal sural SNAP amplitude) was applied, 172 of the initial 312 patients were reclassified into the DPN group, and 140 into the non-DPN group. Using the same PSM protocol (with identical covariates), 142 well-matched pairs (total N = 284) were obtained, with all baseline covariates remaining well-balanced after matching (all Standardized Mean Differences < 0.1).

In this sensitivity analysis cohort, the primary findings remained unchanged. Glycemic variability parameters were significantly higher in the DPN group compared to the non-DPN group (MAGE: 6.7 ± 2.0 vs. 4.8 ± 1.7 mmol/L, P < 0.001; CV: 35.8 ± 8.5% vs. 28.5 ± 7.3%, P < 0.001). In multivariable linear regression adjusted for matched covariates, smoking, and statin use, both MAGE (β = -0.35, 95% CI: -0.49 to -0.21, P < 0.001) and CV (β = -0.29, 95% CI: -0.43 to -0.15, P < 0.001) persisted as significant independent negative predictors of the composite NCV Z-score. ROC analysis yielded optimal discriminatory thresholds highly consistent with the main analysis (MAGE ≥5.7 mmol/L, AUC = 0.82; CV ≥32.0%, AUC = 0.79). These results confirm that the strong association between glycemic variability and DPN is robust and not dependent on the specific motor nerve-based electrophysiological diagnostic criterion used in our primary analysis.

## 4 Discussion

This study provides robust clinical evidence from a propensity score-matched cohort supporting the hypothesis that glycemic variability is an independent and significant risk factor for diabetic peripheral neuropathy in patients with type 2 diabetes. Our principal findings are fourfold. First, after meticulous matching for key confounders including age, diabetes duration, and HbA1c, GV parameters (MAGE, CV, and SD) remained markedly elevated in patients with DPN compared to their non-DPN counterparts, indicating an association with DPN presence. Second, and crucially for assessing severity, multivariable linear regression within this matched cohort demonstrated that both MAGE and CV were independent negative predictors of the composite NCV Z-score. Since reduced NCV is a well-established electrophysiological correlate of DPN severity, this result provides direct evidence for an independent association between higher glycemic variability and more severe neuropathy. Third, mediation analysis revealed that the neurotoxic effect of GV is partly channeled through a pro-inflammatory state, with IL-6 and TNF-α collectively mediating approximately one-third of GV’s total effect on nerve function. Fourth, concomitant neurotrophic dysregulation was observed, characterized by depleted NGF levels in patients with high GV.

Our results align with and extend a growing body of international literature. The independent association between GV and DPN corroborates findings from recent studies. For instance, a cross-sectional analysis by Firouzabadi et al. demonstrated that MAGE was an independent predictor of DPN after multivariable adjustment (18). Similarly, a prospective study by Feldman et al. found that GV metrics derived from CGM predicted the development of neuropathic symptoms over time, whereas HbA1c did not (22). The novelty of our work lies in the application of rigorous propensity score matching, which strengthens causal inference by minimizing selection bias and creating highly comparable groups—a methodological advancement over many previous observational studies. The persistence of a strong GV-DPN association post-matching underscores GV’s potential role as an intrinsic pathogenic factor, not merely a proxy for worse overall glycemic control.

Our study reinforces the growing consensus that DPN pathogenesis in T2DM is multifactorial and extends beyond chronic hyperglycemia. A compelling body of evidence suggests that neuropathy in T2DM may represent a distinct entity from that in type 1 diabetes, with a fundamentally different risk profile. Notably, while intensive glucose control, as measured by HbA1c, has a profound preventive effect on neuropathy in type 1 diabetes, its benefit in T2DM is demonstrably more modest, underscoring the significant contribution of other factors (23). Epidemiologic and laboratory studies consistently implicate components of the metabolic syndrome—including obesity, hypertension, dyslipidemia, and insulin resistance—as critical drivers of nerve injury in T2DM, likely operating through shared pathways of vascular dysfunction, inflammation, and lipotoxicity. Furthermore, the relationship between glucose control and neuropathy may not be uniform across all T2DM patients. Recent evidence using advanced imaging indicates a *diametrical* effect: in patients with established DPN, higher HbA1c is associated with increased nerve microvascular permeability (a marker of damage), whereas in those without DPN, the relationship is inverse (24). This heterogeneity suggests that the pathophysiological implications of hyperglycemia differ depending on the disease stage and underlying susceptibility. Collectively, these observations argue for a paradigm shift beyond HbA1c-centric management. They highlight the need to identify more precise, dynamic, and potentially stage-specific metabolic risk markers. Our investigation into GV is situated within this context. We posit that acute glucose oscillations represent a distinct and potent metabolic stressor—one that is invisible to HbA1c—and may serve as a crucial missing link in understanding DPN risk and progression in the complex milieu of T2DM.

The dose-response relationship we observed adds considerable weight to the argument for causality. The stepwise decline in nerve conduction velocity across increasing MAGE tertiles suggests a graded and cumulative detrimental effect of glucose oscillations on peripheral nerves. This finding is consistent with the concept of “metabolic memory” or “legacy effect,” where prior glycemic exposure patterns can induce lasting epigenetic and functional changes in vulnerable tissues (25). From a mechanistic standpoint, intermittent hyperglycemia may induce more severe endoplasmic reticulum stress, mitochondrial dysfunction, and impaired autophagy in neurons and Schwann cells than stable hyperglycemia, ultimately overwhelming cellular repair mechanisms (26, 27). Our integrated biomarker data provide clinical support for specific intermediary pathways.

The inflammatory mediation pathway uncovered represents a significant contribution to the field. While systemic inflammation is a recognized component of diabetic neuropathy pathogenesis (15), its specific role as a mediator between GV and nerve damage has been quantitatively underexplored in clinical studies. Our analysis indicates that ~32% of GV’s detrimental effect on NCV operates via elevated IL-6 and TNF-α. This is mechanistically plausible, as experimental models show that oscillating glucose potently activates the NLRP3 inflammasome in vascular endothelial cells and dorsal root ganglia, leading to caspase-1 activation and the release of pro-inflammatory cytokines IL-1β and IL-18 (14). TNF-α is a known direct inducer of neuronal apoptosis and an inhibitor of axonal regeneration (17). Therefore, therapeutic strategies aimed at reducing GV may confer neuroprotection partly by dampening this GV-triggered inflammatory cascade.

The observed neurotrophic dysregulation, particularly the inverse correlation between GV and NGF, provides another compelling mechanistic link. NGF is crucial for the maintenance, survival, and regenerative capacity of small sensory nerve fibers, which are often affected early in DPN (16). Our finding of lower NGF levels in the highest GV tertile suggests that glucose instability may impair the synthesis, axonal transport, or signaling of key neurotrophic factors. The U-shaped relationship observed for IGF-1 is intriguing and warrants further investigation; it may represent a compensatory hormonal response to metabolic stress or reflect altered tissue-specific clearance.

The ROC analysis yielded clinically actionable thresholds (MAGE ≥5.8 mmol/L, CV ≥32.5%) for DPN risk stratification. The superior discriminative power of GV indices over HbA1c alone suggests that incorporating CGM-derived GV metrics into routine clinical practice could significantly refine DPN risk assessment. This is particularly relevant for overcoming therapeutic inertia, as demonstrating a direct link between GV and a tangible, disabling complication may motivate both patients and clinicians to adopt or intensify therapies known to smooth glucose profiles, such as GLP-1 receptor agonists, SGLT2 inhibitors, or advanced insulin delivery systems.

Our findings support an integrated pathogenic framework in which GV is not merely an adjunct to chronic hyperglycemia, but a central driver that concurrently and synergistically amplifies both the metabolic and vascular pathways implicated in DPN (1, 2). In this model, acute glucose excursions initiate a dual assault. First, each glycemic spike generates excessive ROS within neurons and Schwann cells, exceeding the oxidative stress induced by stable hyperglycemia (11, 12). This direct metabolic insult disrupts mitochondrial function, bioenergetics, and neurotrophic support (e.g., NGF depletion). Second, GV potently activates a systemic inflammatory response (elevated IL-6 and TNF-α, as shown here), which, together with oxidant spillover, targets the vasa nervorum (15). This leads to endothelial dysfunction, impaired endoneurial blood flow, and hypoxia (28). The consequence is a vicious, self-reinforcing cycle: the axon is metabolically compromised from within, while its microvascular supply fails from without. The resulting hypoxia further exacerbates mitochondrial dysfunction and oxidative stress, accelerating axonal degeneration and demyelination 3434. This unified mechanism explains the particular potency of GV—it delivers a coordinated attack on both the neuron’s intrinsic health and its extrinsic vascular support. Our mediation analysis, indicating that systemic inflammation accounts for approximately one-third of GV’s detrimental effect on nerve conduction, provides direct clinical validation for the critical intermediary role of this inflammatory-vascular bridge (15, 28). Therefore, GV likely contributes to DPN by orchestrating a pathogenic synergy between metabolic toxicity and microvascular failure. This perspective underscores that therapeutic strategies aimed at stabilizing glucose may confer neuroprotection by mitigating injury across both key domains.

Key strengths of this study include the use of PSM to enhance comparability, objective electrophysiological confirmation of DPN, comprehensive GV assessment via standardized CGM, and the integration of mechanistic serum biomarkers. Several limitations must be acknowledged. First and foremost, the retrospective and cross-sectional nature of our study design precludes any definitive causal inference. Although propensity score matching strengthened comparability and multivariable regression adjusted for confounders, glycemic variability and DPN status were assessed concurrently. Therefore, we cannot determine the temporal sequence: whether elevated glycemic variability precedes and contributes to the development of DPN, or whether the presence of DPN (with its associated changes in autonomic function, physical activity, or disease management) leads to greater glycemic instability. It is plausible that the relationship is bidirectional. Thus, our findings demonstrate a strong and independent association, but not causation. Future prospective, longitudinal cohort studies with serial CGM and standardized neuropathy assessments are essential to establish whether GV is a predictor of DPN incidence or progression over time. Moreover, and importantly, the controlled inpatient environment during which CGM data were collected represents a significant limitation. Patients were hospitalized for routine diabetes management, workup, or stabilization of comorbid conditions. While this ensured standardized meal timing and composition, as well as reduced physical activity, it likely resulted in a more regulated and potentially attenuated glycemic profile compared to free-living conditions. The 72-hour CGM data, therefore, may not fully capture the amplitude and frequency of glucose excursions that occur in daily life with variable diet, exercise, stress, and adherence patterns. Consequently, the association between glycemic variability and DPN observed in our study might be an underestimation of the true effect size in an outpatient setting. Future prospective studies utilizing CGM in ambulatory patients over longer periods are needed to validate our findings in a more ecologically valid context. Additionally, our mechanistic analyses using serum biomarkers and mediation modeling should be interpreted with caution due to sample size and selection constraints. The biomarker subcohort (n=160), while representative in terms of matched baseline characteristics, was limited to patients with available biobanked samples. This selection based on sample availability, though not systematically biased in known clinical variables, could introduce unmeasured confounding and limits the generalizability of the biomarker findings. Consequently, the mediation analysis suggesting that inflammation accounts for approximately 32% of GV’s effect on NCV is hypothesis-generating and requires validation in larger, prospectively designed cohorts where biomarkers are systematically collected for all participants. The modest sample size for these analyses also precluded more complex modeling or exploration of other potential mediators. In addition, the lack of small fiber neuropathy assessment (e.g., via skin biopsy or corneal confocal microscopy) is a notable gap, as small fibers are often affected earlier in the disease process. Finally, regarding our electrophysiological diagnostic criteria. Diabetic peripheral neuropathy typically manifests with sensory abnormalities earlier than motor conduction slowing. Our primary diagnostic criterion relied on abnormal MNCV in at least two nerves. While this approach ensured the identification of definite, objective nerve injury and provided a standardized metric for severity (composite NCV Z-score), it may have inadvertently excluded patients with early-stage DPN whose primary electrophysiological abnormality was reduced sensory nerve action potential amplitude. Notably, our pre-planned sensitivity analysis—which employed a broader diagnostic criterion incorporating sural SNAP amplitude—yielded fully consistent results, underscoring the robustness of our primary conclusions. Nevertheless, the potential for selection bias towards patients with more advanced or motor-predominant neuropathy in the primary analysis cannot be entirely ruled out. This may limit the generalizability of our findings to the earliest, purely sensory stages of DPN. Future studies should employ comprehensive diagnostic criteria that explicitly include sensory nerve parameters to capture the full spectrum of the disease.

## 5 Conclusion

In conclusion, this propensity score-matched cohort study provides compelling evidence that glycemic variability is an independent and clinically significant contributor to diabetic peripheral neuropathy, with systemic inflammation serving as a key mediating pathway. Our findings advocate for a paradigm shift in diabetes management—from a singular focus on HbA1c toward a comprehensive, dual-goal strategy that equally emphasizes achieving glycemic stability. Future prospective, multicenter, interventional studies are urgently needed to determine whether therapeutic strategies specifically designed to reduce GV can prevent or retard the progression of DPN more effectively than conventional glycemic control alone.

## Data Availability

The raw data supporting the conclusions of this article will be made available by the authors, without undue reservation.
